# Brucellar Spondylitis with Epidural Abscess and Lumbosacral Radiculitis Mimicking Lumbar Disc Herniation

**DOI:** 10.1590/0037-8682-0460-2025

**Published:** 2026-02-06

**Authors:** Handan Alay, Elif Gözgeç

**Affiliations:** 1 Ataturk University, Faculty of Medicine, Department of Infection Diseases and Clinical Microbiology, Erzurum, Turkey.; 2 Ataturk University, Faculty of Medicine, Department of Radiology, Erzurum, Turkey.

A 48-year-old man presented with progressive low back pain from last two months, radiating to the right hip and thigh. He had previously been treated with nonsteroidal anti-inflammatory drugs for suspected lumbar disc herniation, but showed no improvement. He had no history of consumption of unpasteurized milk or dairy products. However, he reported that he was actively involved in animal care as a hobby. Laboratory tests showed a positive Rose Bengal test result, a Wright agglutination titer of 1:1280, Brucella IgM of 0.32, and IgG of 3.7, whereas blood cultures were negative. The patient was treated with doxycycline (200 mg/day) and streptomycin (1 g/day).

On day 10, the patient developed worsening radicular pain radiating to the right lower extremities and buttocks. Magnetic resonance imaging revealed L5-S1 spondylitis with a 12 × 26 × 32 mm peripherally enhancing epidural abscess and right neural foraminal enhancement, consistent with radiculitis **(** Figure 1 **)**. Antimicrobial treatment was intensified with doxycycline, rifampin, and ceftriaxone. Significant clinical improvement was observed within one week.

**FIGURE 1:**
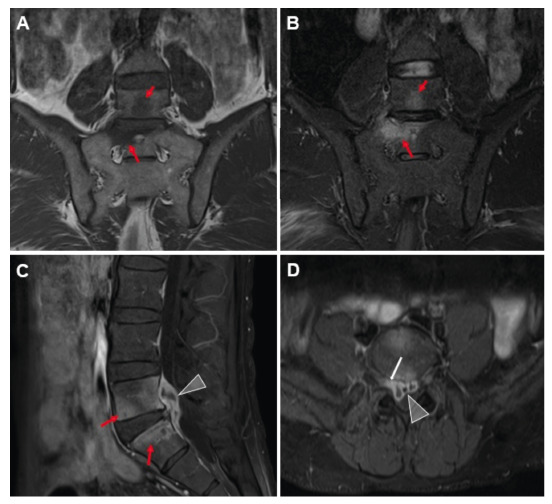
On magnetic resonance images, the L5 and S1 vertebral bodies appear hypointense on T1-weighted coronal images **(A)** and hyperintense on T2-weighted coronal images **(B)** (arrows). Post-contrast T1-weighted sagittal **(C)** and axial **(D)** images show increased contrast enhancement in the vertebrae (red arrows), a peripherally enhanced abscess focus in the epidural space (arrowheads), and right nerve root enhancement (white arrow).

Abscess formation with atypical involvement may occur because of brucella spondylitis^1^. Brucellar spondylodiscitis involving the lower back and lumbar region is often misdiagnosed as sciatica or herniated discs owing to a similar presentation^2^. 

## References

[1] Yuksel KZ, Senoglu M, Yuksel M, Gul M (2006). Brucellar spondylo-discitis with rapidly progressive spinal epidural abscess presenting with sciatica. Spinal Cord.

[2] Alay H, Karadağ MK, Çankaya BY (2023). Brucellar Cervical Spondylodiscitis Complicated by Epidural Abscess and Neurobrucellosis. Rev Soc Bras Med Trop.

